# The Gradient of Immune/Inflammatory Response and COVID-19 Prognosis with Therapeutic Implications

**DOI:** 10.3389/fimmu.2021.739482

**Published:** 2021-10-29

**Authors:** Ming Zheng

**Affiliations:** ^1^ Institute of Military Cognition and Brain Sciences, Academy of Military Medical Sciences, Beijing, China; ^2^ Beijing Institute of Basic Medical Sciences, Beijing, China

**Keywords:** COVID-19, SARS-CoV-2, CRP (C-reactive protein), lymphocyte, prognosis, inflammation

## Introduction

As of October 8, 2021, there were 236,599,025 confirmed cases and 4,831,486 deaths attributed to COVID-19 (https://covid19.who.int/). The COVID-19 pandemic puts high pressure on medical resources worldwide (1). To reduce the clinical burden and casualties caused by COVID-19, it is urgent to investigate which patients are at high risk and thus in imminent need of critical care services. Hemogram characteristics hold great promise for predicting the clinical outcomes of COVID-19 (2). However, there is still a scarcity of data modeling the gradient of hemogram parameter as a continuous risk predictor of COVID-19 prognosis. Through leveraging the hemogram data of 485 COVID-19 patients and analyzing the pointwise prognostic values across continuous biological and clinical indices, we explored the nonlinear relationship between the continuous hemogram values and their prognostic associations with COVID-19. The immune/inflammatory markers — lymphocytes (%), neutrophils (%), and high-sensitivity C-reactive protein (hs-CRP, mg/l) — were significantly associated with the overall survival (OS) of COVID-19 patients in dose-dependent manners. This study shed light on the dose-dependent effects of immune and inflammatory responses on the prognosis of COVID-19 patients, with important implications in immunomodulation and anti-inflammatory therapies for COVID-19. Moreover, our analytical framework will help better delineate clinical predictors for not only COVID-19 but also other human diseases and facilitate the discovery of biomarker and disease mechanisms.

## The Analysis Framework of Nonlinear Prognostic Relevance and Its Application in the Hemogram Characteristics and Prognosis of COVID-19 Patients

In this study, we analyzed the hemogram characteristics in 485 COVID-19 patients collected in Tongji Hospital (Wuhan, China) from a previous study (2), as shown in **Supplementary Figure 1**. For each individual patient, if the hemogram was tested more than once, the median value was calculated for further analysis. All the hemogram values were normalized by Min-Max Scaler (MMS). Next, using a statistical approach of smooth HR (3) as previously reported (4), we evaluated the nonlinearly prognostic values across the gradients of continuous hemogram values. Based on Cox proportional hazard regression, the pointwise hazard ratio (HR) was calculated for each hemogram value, taking the median hemogram value as the reference (**Supplementary Figure 2**). The prognostic associations between hemogram parameters and survival were assessed by both univariate and multivariate analyses. To rule out the effect of outliers, truncation was performed to remove hemogram values below 2.5th and above 97.5th percentiles. The standard deviations (SDs) of pointwise HRs were used for ranking the prognostic effect of different hemogram parameters, showing how much the gradient of continuous hemogram values would affect its prognostic associations.

Next, we used the t-distributed stochastic neighbor embedding (t-SNE) (5) to conduct the dimensionality reduction of top-ranked hemogram parameters associated with prognosis and paint the hemogram portraits of COVID-19 patients in three-dimensional space.

## Significant Prognostic Associations of Age and Sex in COVID-19

**Figure 1:**
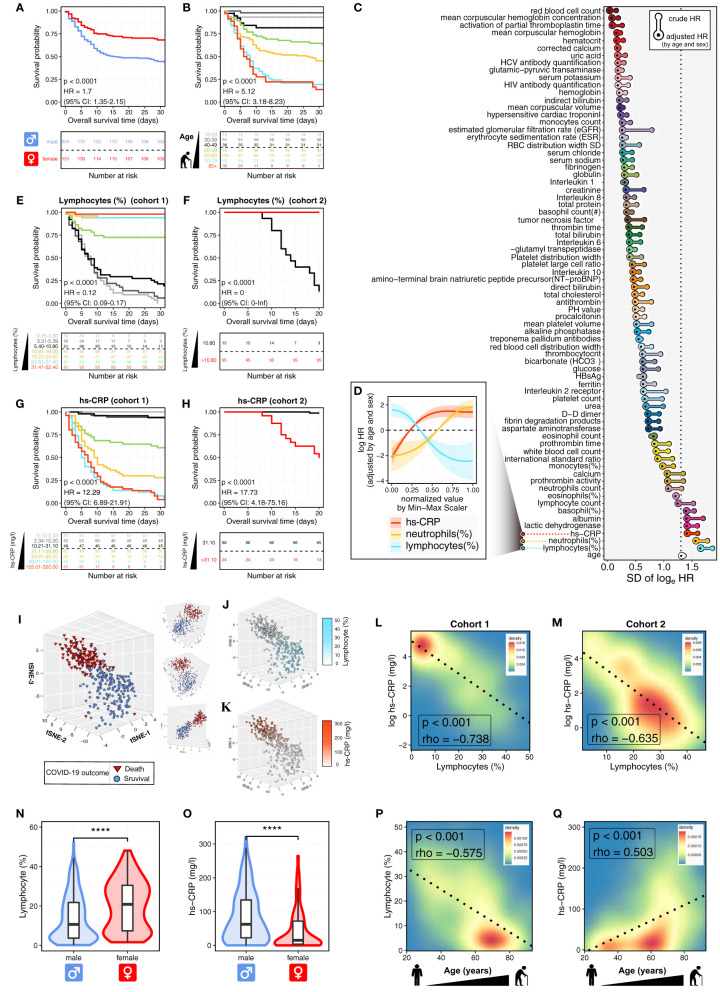
Prognosis-associated hemogram parameters in COVID-19 patients. **(A, B)** The Kaplan-Meier curves show the overall survival (OS) of 375 COVID-19 patients (cohort 1) according to sex **(A)** and age **(B)**. Log-rank *p* values and HRs (95% CIs) show the comparisons between males and females **(A)**, cases with age < 50 and ≥ 50 years **(B)**. CI, confidence interval; HR, hazard ratio. The bottom panel shows the number of patients at risk every five days. **(C)** The waterfall plot shows the standard deviations (SDs) of log HRs, both crude and adjusted by age and sex. The pointwise HRs were calculated by fitting Cox proportional hazard models for the continuous hemogram values, taking the median hemogram value as the reference. Next, hemogram parameters were ranked by the SDs of adjusted HRs. The top three hemogram parameters were immune/inflammatory variables, including lymphocytes (%), neutrophils (%), and high-sensitivity C-reactive protein (hs-CRP, mg/l). **(D)** The continuous log-HR curves (solid line) with 95% CIs (shading) show the association of immune/inflammatory parameters with OS in COVID-19 patients (cohort 1). The age- and sex-adjusted HRs were fitted based on Cox proportional hazards regression as described above. The x-axis shows normalized values of immune/inflammatory parameters by Min-Max Scaler (MMS). **(E, F)** The Kaplan-Meier curves show OS of COVID-19 patients according to lymphocytes (%) in cohort 1 **(E)** and cohort 2 **(F)**. Log-rank *p* values and HRs (95% CIs) show the comparison between patients with lymphocytes (%) ≤ 10.80 and > 10.80. The bottom panel shows the number of patients at risk every five days. **(G, H)** The Kaplan-Meier curves show OS of COVID-19 patients according to hs-CRP (mg/l) in cohort 1 **(G)** and cohort 2 **(H)**. Log-rank *p* values and HRs (95% CIs) show the comparison between patients with hs-CRP (mg/l) ≤ 31.10 and > 31.10. The bottom panel shows the number of patients at risk every five days. **(I)** The t-SNE plot shows the projection of the top 15 prognosis-associated hemogram parameters into a 3D map. Each dot represents an individual patient. The dot color and shape represent the COVID-19 outcomes of death (red triangle) and survival (blue circle). **(J, K)** The t-SNE plot shows the immune/inflammatory response in COVID-19 patients. The dot shape represents the COVID-19 outcomes of death (triangle) and survival (circle), with color coding according to lymphocytes (%) **(J)** and hs-CRP (mg/l) **(K)**. **(L, M)** The contour plots show the Spearman correlations between lymphocytes (%) and hs-CRP (mg/l) in cohort 1 **(L)** and cohort 2 **(M)**. The dashed line represents the linear regression line. **(N, O)** The violin plot shows the distributions of lymphocytes (%) **(N)** and hs-CRP (mg/l) **(O)** in the subgroups defined by patients’ sex. The box plot shows the distribution from the 25th to the 75th percentile. The line inside the box plot represents the median. The whiskers reflect the 95% confidence intervals. The results were considered statistically significant when *p* < 0.0001 (****) using the Student’s t-test. **(P, Q)** The contour plots show the correlations of COVID-19 patients’ age with lymphocytes (%) **(P)** and hs-CRP (mg/l) **(Q)**. The dashed line represents the linear regression line. The correlation was measured by the Spearman correlation coefficient (rho).

It is reported that COVID-19 disproportionally causes severe and fatal outcomes in male and older populations (6). In this study, we tested and confirmed the quality of the COVID-19 cohort by analyzing the associations of sex and age with the overall survival (OS) of COVID-19 patients (**Figures 1A, B**). Male patients had poor outcomes with a median OS time of 15.5 days (95% CI, 11 to NR (not reached)) and an OS rate of 43.8%. By contrast, female patients had an OS rate of 68.2% (**Figure 1A**). Notably, we observed that since the age of 30 years, there was a stepwise reduction in OS for each decade increase in patients’ age. Both the 18-29 and 30-39 age groups had only one deceased patient, respectively; and their OS rates were as high as 93.3% and 98.0%. Patients aged more than 50 years had particularly poor outcomes with a median OS time of 13 days (95% CI, 9 to 26) and an OS rate of 39.1%. By contrast, patients aged less than 50 years had an OS rate of 91.3% (**Figure 1B**). The sex- and age-related prognostic associations in COVID-19 were consistent with previously reported data. Thus, age and sex were selected as covariates for multivariate analysis in this study.

## The Gradient of Immune/Inflammatory Response Predicts the Prognosis of COVID-19 Patients

To quantify the nonlinear prognostic associations across the continuous hemogram values, we calculated the pointwise hazard ratios (HRs) for each hemogram value based on the Cox proportional hazard regression (**Supplementary Figure 2**). We observed remarkable differences between the crude and adjusted HRs for almost all hemogram parameters (**Figure 1C**), showing the importance of controlling for covariates of patients’ sex and age. Moreover, the HR curves successfully captured the nonlinear relationship between the HR for OS and the continuous values of different hemogram parameters (**Figure 1D**; **Supplementary Figure 3**).

After the hemogram parameters were ranked by the standard deviations (SDs) of sex- and age-adjusted HRs, we observed that the top three hemogram parameters were immune/inflammatory parameters, including lymphocytes (%), neutrophils (%), and high-sensitivity C-reactive protein (hs-CRP, mg/l) (**Figure 1C**). Notably, the above immune/inflammatory parameters were significantly associated with OS in dose-dependent manners (**Figure 1D**). As shown in **Supplementary Figure 2**, the HR curve with 95% CI has a straightforward interpretation — 95% CI above or below 0 is equivalent to a two-sided *p* < 0.05.

Next, Kaplan-Meier curves showed that the immune/inflammatory parameters were strongly predictive of OS (**Figures 1E–H**; **Supplementary Figure 4**). Patients in cohort 1 were divided equally into seven groups according to hemogram values. For each decrease in lymphocytes (%), there was a stepwise reduction in OS (**Figure 1E**). COVID-19 patients with lymphocytes (%) ≤ 10.80% had particularly poor outcomes with median OS times of 7 days (95% CI, 6 to 9) and 14 days (95% CI, 12 to 20) in cohort 1 and 2, with the OS rates of 7.8% and 13.3%, respectively. By contrast, patients with lymphocytes (%) > 10.80% had OS rates of 85.1% and 100% in cohort 1 and 2, respectively (**Figures 1E, F**).

Next, we found that hs-CRP was also remarkably associated with OS in a dose-dependent manner (**Figure 1G**). COVID-19 patients with hs-CRP > 31.10 (mg/l) had inferior outcomes with median OS times of 9 days (95% CI, 7 to 10) and 20 days (95% CI, 15 to NR) and OS rates of 25.0% and 50.0% in cohort 1 and 2, respectively. By contrast, patients with hs-CRP ≤ 31.10 (mg/l) had OS rates of 96.0% and 98.8% in cohort 1 and 2, respectively (**Figures 1G, H**). The above results indicated the dose-dependent effect of immune/inflammatory parameters on COVID-19 prognosis. These dose-dependent effects implied that the gradient of immune/inflammatory response might play a causal role in COVID-19 severity, which could be an early detectable laboratory test before disease progression occurs.

## Hemogram Portraits of COVID-19 Patients and the Inverse Relationship Between Immune and Inflammatory Responses

At last, we used t-SNE to project the top 15 prognosis-associated hemogram parameters identified in **Figure 1C** into a three-dimensional (3D) space. As shown in **Figures 1I–K**, each dot represents an individual COVID-19 patient. Notably, we observed the distinct clustering between deceased and survived COVID-19 patients (**Figure 1I**). Furthermore, compared to the deceased COVID-19 patients, the COVID-19 survivors showed a specific immune/inflammatory phenotype of high lymphocytes (%) and low hs-CRP (mg/l) (**Figures 1J, K**). Next, we discovered that lymphocytes (%) were significantly inversely correlated with hs-CRP (mg/l) in both cohort 1 (*p* < 0.001, Spearman’s rho = -0.738, **Figure 1L**) and cohort 2 (*p* < 0.001, Spearman’s rho = -0.635, **Figure 1M**).

## Sex- and Age-Related Differences in the Immune and Inflammatory Responses of COVID-19 Patients

Next, we sought to explore the sex- and age-related differences in the immune and inflammatory responses of COVID-19 patients. The males had significantly 0.96-fold lower lymphocytes (%) and 3.15-fold higher hs-CRP (mg/l) than those of females (*p* < 0.0001; **Figures 1N, O**). Additionally, the increased age was remarkably associated with decreased lymphocytes (%) and increased hs-CRP (mg/l) (*p* < 0.001; **Figures 1P, Q**). It is worth noting that the patients aged more than 50 years had significantly 2.19-fold lower lymphocytes (%) and 4.97-fold higher hs-CRP (mg/l) than those aged less than 50 years. The above results showed the markedly decreased immune response and increased inflammation in male and older patients with COVID-19, indicating the intricate interplay between the patients’ sex, age, and the immune/inflammatory response to COVID-19.

## Discussion

Hemogram characteristics represent promising candidates as predictive and prognostic biomarkers. Despite recent intensive efforts to leverage the hemogram of COVID-19, different studies used different thresholds for stratifying the continuous hemogram values, leading to inconsistent results and ambiguous translational implications. To address these issues and gain new insights into the hemogram features and their prognostic associations with COVID-19, we integrated the hemogram characteristics of COVID-19 within the analysis framework of nonlinear prognostic relevance. Thus, this study differs from previous efforts, which were not limited to a single threshold of hemogram values, and presented the nonlinearly prognostic associations in a dose-dependent manner.

Based on this novel analysis framework, we identified the dose-dependent effect of immune/inflammatory response on patients’ survival. These findings are consistent with the previously reported studies that the progression of COVID-19 is related to the aberrant immune/inflammatory response (7, 8). Moreover, the aggressive inflammatory response inflicts multi-organ damage leading to organ failure in COVID-19 patients (9). Additionally, it is well acknowledged that an aberrant inflammation suppresses the immune response, thus favoring viral infection. In this study, using the powerful analytical tool of t-SNE dimensional reduction analysis, we constructed a 3D projection of the hemogram characteristics in COVID-19 patients, revealing that the survived and deceased patients had distinct hemogram portraits which were associated with the significantly inversely related immune and inflammatory gradients. This finding fits the model of the suppression of immune response by inflammation. Therefore, we proposed that these immune/inflammatory parameters, as early detectable laboratory tests, could be used to predict the clinical outcome of COVID-19 before disease progression occurs.

Furthermore, the dose-dependent effect of immune/inflammatory gradient on COVID-19 prognosis indicates the causal role of immune/inflammatory response in COVID-19 severity, implying immunomodulation and anti-inflammatory therapies as potential strategies to slow or reverse disease progression. Recently, the emerging immunomodulation and anti-inflammatory therapies have yielded unprecedented promising in COVID-19 treatment (10, 11). Many ongoing trials have been conducted to investigate the potential effects of immunomodulation and anti-inflammatory therapies in COVID-19 patients (12). For such therapies, the immune/inflammatory hemogram portraits could also provide candidate biomarkers for better understanding the treatment response or failure. Moreover, the analytical framework presented herein is also amenable as a useful approach for future studies, including the investigation of prognostic biomarkers and effectors with continuous values and ambiguous threshold and, meanwhile, the assessment of both univariate and multivariate interaction between clinical indices and disease outcomes.

## Author Contributions

MZ conceived the project, developed the method, conducted data analysis, and wrote the manuscript.

## Funding

This project was supported by the National Natural Science Foundation of China (32100739) to MZ.

## Conflict of Interest

The author declares that the research was conducted in the absence of any commercial or financial relationships that could be construed as a potential conflict of interest.

## Publisher’s Note

All claims expressed in this article are solely those of the authors and do not necessarily represent those of their affiliated organizations, or those of the publisher, the editors and the reviewers. Any product that may be evaluated in this article, or claim that may be made by its manufacturer, is not guaranteed or endorsed by the publisher.

## Supplementary Material

Supplementary Figure 1 Informative characteristics of laboratory tests in COVID-19 patients. SE, standard error; SV, coefficient of variation.

Supplementary Figure 2 Diagram of prognostic analysis of continuous hemogram values. Based on Cox proportional hazard regression, the point wise hazard ratio (HR) was calculated for each of the continuous hemogram values, taking the median hemogram value as the reference. The associations between hemogram parameters and survival were assessed by both univariate and multivariate Cox regression. Next, Kaplan-Meier curves were plotted to show the overall survival of COVID-19 patients according to hemogram values.

Supplementary Figure 3 Continuous log-HR curves (solid line) with 95% CIs (shading) show the association of lactate dehydrogenase (LDH), basophil (%), albumin with OS in COVID-19 patients. The age- and sex-adjusted HRs were fitted based on Cox proportional hazards regression. The x-axis shows the normalized hemogram values by Min-Max Scaler (MMS).

Supplementary Figure 4 The Kaplan-Meier curves show OS of COVID-19 patients according to neutrophils (%) in cohort 1. Log-rank p values and HRs (95% CIs) show the comparison between patients with neutrophils (%) ≤ 72.50 and > 72.50. The bottom panel shows the number of patients at risk every five days.
