# Supplementary material for: The Gradient of Immune/Inflammatory Response and COVID-19 Prognosis with Therapeutic Implications
Source: Front Immunol. 2021 Oct 29;12:739482. doi: 10.3389/fimmu.2021.739482 (PMC8586492; doi:10.3389/fimmu.2021.739482)
Supplement: Supplementary Figure 1 — Informative characteristics of laboratory tests in COVID-19 patients. SE, standard error; SV, coefficient of variation. [file Image_1.pdf]

Name	Mean	SE	SV
lymphocytes(%)	16.54	0.66	0.75
neutrophils(%)	75.95	0.86	0.21
hs-CRP	68.70	3.82	1.04
lactic dehydrogenase	456.19	18.37	0.76
albumin	33.13	0.29	0.17
basophil(%)	0.22	0.01	0.86
lymphocyte count	1.09	0.12	2.10
eosinophils(%)	0.65	0.05	1.52
neutrophils count	7.30	0.29	0.75
prothrombin activity	82.59	1.06	0.24
calcium	2.11	0.01	0.06
monocytes(%)	6.55	0.21	0.61
international standard ratio	1.21	0.02	0.30
white blood cell count	9.66	0.58	1.13
prothrombin time	15.58	0.33	0.40
eosinophil count	0.04	0.00	1.46
aspartate aminotransferase	46.27	5.11	2.08
fibrin degradation products	47.68	4.26	1.27
D-D dimer	6.32	0.46	1.33
urea	8.55	0.43	0.96
platelet count	193.47	5.07	0.49
Interleukin 2 receptor	934.34	54.92	0.86
ferritin	1486.52	264.50	2.60
HBsAg	8.43	2.61	5.13
glucose	8.29	0.22	0.51
bicarbonate (HCO3-)	22.91	0.20	0.16
thrombocytocrit	0.21	0.00	0.40
red blood cell distribution width	12.98	0.09	0.13
treponema pallidum antibodies	0.13	0.05	5.84
alkaline phosphatase	81.50	2.57	0.59
mean platelet volume	10.87	0.05	0.09
procalcitonin	0.85	0.20	4.14
PH value	6.45	0.04	0.10
antithrombin	87.86	1.14	0.18
total cholesterol	3.67	0.05	0.24
direct bilirubin	8.29	1.12	2.55
amino-terminal brain natriuretic peptide precursor(NT-proBNP)	2550.34	487.34	3.12
Interleukin 10	13.92	3.51	3.70
platelet large cell ratio	31.52	0.43	0.25
Platelet distribution width	12.94	0.14	0.20
gamma-glutamyl transpeptidase	49.72	3.31	1.26
Interleukin 6	107.49	29.72	4.08
total bilirubin	14.94	1.45	1.83
thrombin time	17.50	0.30	0.29
tumor necrosis factor alpha	11.62	0.75	0.95
basophil count(#)	0.02	0.00	0.79
total protein	66.34	0.32	0.09
Interleukin 8	66.78	21.30	4.69
creatinine	104.03	7.23	1.31
Interleukin 1 beta	6.55	0.48	1.08
globulin	33.23	0.26	0.15
