# Supplementary material for: The Gradient of Immune/Inflammatory Response and COVID-19 Prognosis with Therapeutic Implications
Source: Front Immunol. 2021 Oct 29;12:739482. doi: 10.3389/fimmu.2021.739482 (PMC8586492; doi:10.3389/fimmu.2021.739482)
Supplement: Supplementary Figure 2 — Diagram of prognostic analysis of continuous hemogram values. Based on Cox proportional hazard regression, the point wise hazard ratio (HR) was calculated for each of the continuous hemogram values, taking the median hemogram value as the reference. The associations between hemogram parameters and survival were assessed by both univariate and multivariate Cox regression. Next, Kaplan-Meier curves were plotted to show the overall survival of COVID-19 patients according to hemogram values. [file Image_2.pdf]

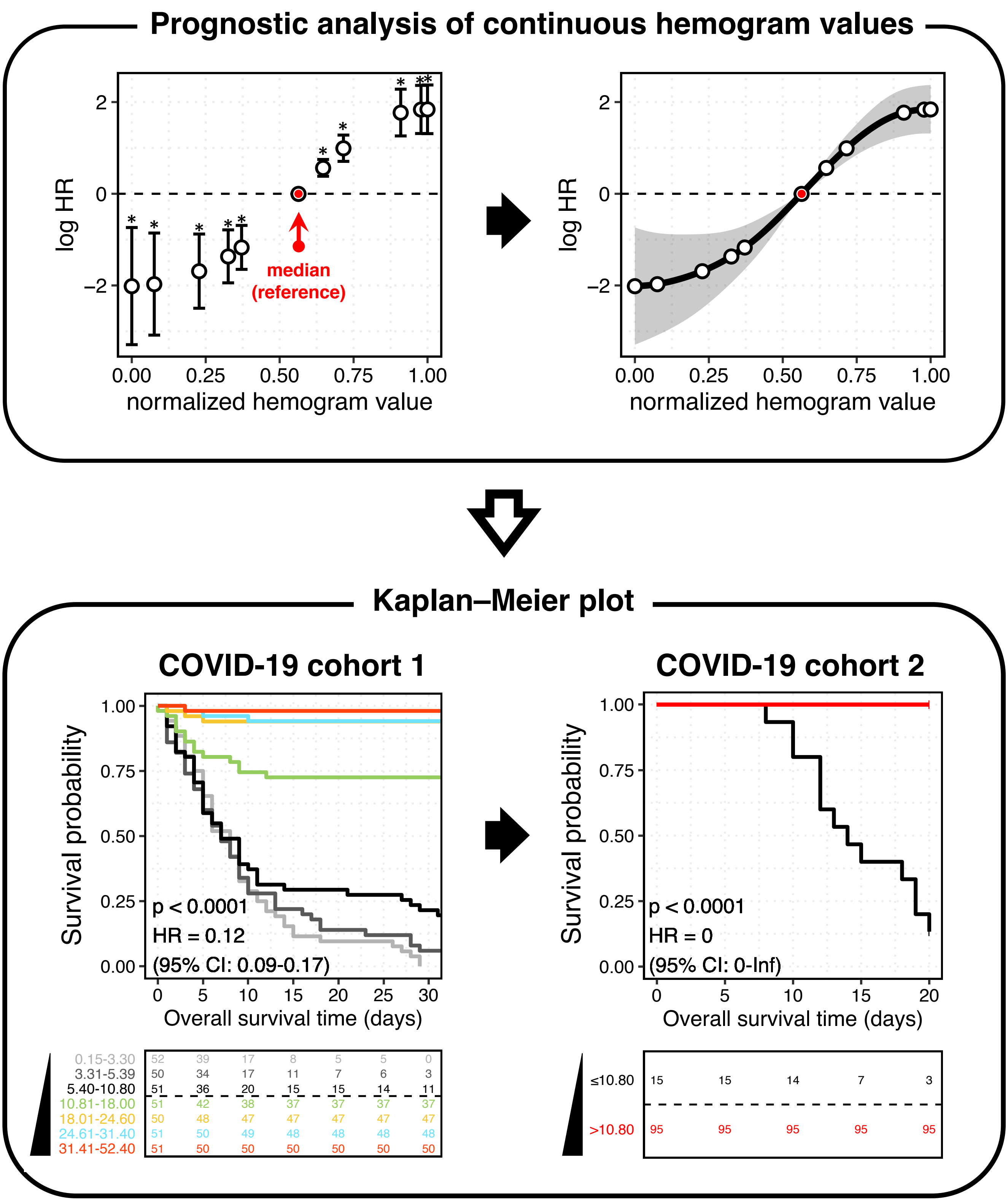

Prognostic analysis of continuous hemogram values
log HR
normalized hemogram value
median
(reference)
Kaplan–Meier plot
COVID-19 cohort 1
COVID-19 cohort 2
Survival probability
Overall survival time (days)
p < 0.0001
HR = 0.12
(95% CI: 0.09-0.17)
HR = 0
(95% CI: 0-Inf)
0.15-3.30
3.31-5.39
5.40-10.80
10.81-18.00
18.01-24.60
24.61-31.40
31.41-52.40
≤10.80
>10.80
