# Supplementary figures and images for: The Gradient of Immune/Inflammatory Response and COVID-19 Prognosis with Therapeutic Implications
Source: Front Immunol. 2021 Oct 29;12:739482. doi: 10.3389/fimmu.2021.739482 (PMC8586492; doi:10.3389/fimmu.2021.739482)

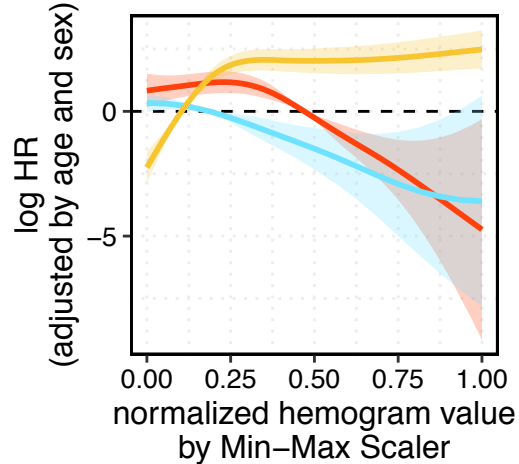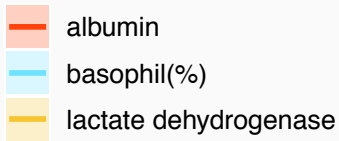

Supplement: Supplementary Figure 3 — Continuous log-HR curves (solid line) with 95% CIs (shading) show the association of lactate dehydrogenase (LDH), basophil (%), albumin with OS in COVID-19 patients. The age- and sex-adjusted HRs were fitted based on Cox proportional hazards regression. The x-axis shows the normalized hemogram values by Min-Max Scaler (MMS). [file Image_3.pdf]

Survival probability

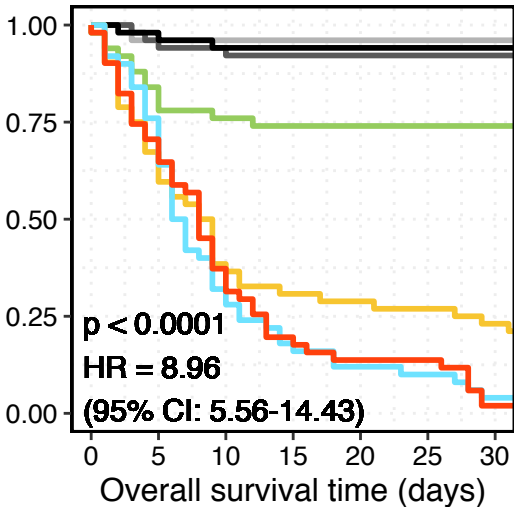

neutrophils (%)

1.80-56.70  
 56.71-65.50  
 65.51-72.50  
 72.51-82.40  
 82.41-90.10  
 90.11-93.70  
 93.71-98.80

|    |    |    |    |    |    |    |
|----|----|----|----|----|----|----|
| 51 | 49 | 49 | 49 | 49 | 49 | 49 |
| 51 | 49 | 48 | 47 | 47 | 47 | 47 |
| 51 | 50 | 48 | 48 | 48 | 48 | 48 |
| 50 | 42 | 38 | 37 | 37 | 37 | 37 |
| 52 | 35 | 20 | 16 | 15 | 14 | 12 |
| 50 | 38 | 16 | 9  | 6  | 5  | 2  |
| 51 | 36 | 19 | 10 | 7  | 7  | 1  |

Supplement: Supplementary Figure 4 — The Kaplan-Meier curves show OS of COVID-19 patients according to neutrophils (%) in cohort 1. Log-rank p values and HRs (95% CIs) show the comparison between patients with neutrophils (%) ≤ 72.50 and > 72.50. The bottom panel shows the number of patients at risk every five days. [file Image_4.pdf]
